# 4-[4,5-Bis(pyridin-2-yl)-1*H*-imidazol-2-yl]phenol monohydrate

**DOI:** 10.1107/S1600536810052062

**Published:** 2010-12-18

**Authors:** Guo-Yong Xiao, Hai-Jun Chi, Peng Lei, Jiang-Long Yu, Zhi-Zhi Hu

**Affiliations:** a School of Chemical Engineering, University of Science and Technology Liaoning, Anshan 114051, People’s Republic of China; b School of Power and Energy Engineering, Shenyang Institute of Aeronautical Engineering, Shenyang, Liaoning, Shenyang 110136, People’s Republic of China

## Abstract

In the title hydrate, C_19_H_14_N_4_O·H_2_O, the dihedral angle between the two pyridine rings is 38.0 (2)°. The dihedral angle between the imidazole and benzene rings is 25.3 (2)°. The crystal structure is stabilized by inter­molecular O—H⋯O, O—H⋯N and N—H⋯O hydrogen bonds.

## Related literature

For early studies of lophine (2,4,5-triphenylimidazole), see: Radziszewsky (1877 ▶). For further synthetic details, see: Nakashima *et al.* (1995 ▶); Kuroda *et al.* (1993 ▶).
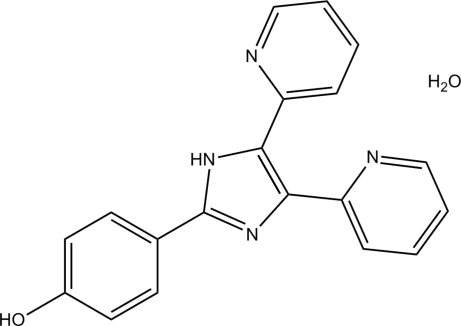


## Experimental

### 

#### Crystal data

C_19_H_14_N_4_O·H_2_O
*M* *_r_* = 332.36
Triclinic, 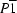

*a* = 8.5875 (17) Å
*b* = 9.0151 (18) Å
*c* = 11.353 (2) Å
α = 77.89 (3)°
β = 69.96 (3)°
γ = 73.66 (3)°
*V* = 786.0 (3) Å^3^
*Z* = 2
Mo *K*α radiation
μ = 0.10 mm^−1^
*T* = 113 K
0.22 × 0.20 × 0.16 mm

#### Data collection

Bruker SMART CCD diffractometer
Absorption correction: multi-scan (*SADABS*; Bruker, 1998 ▶) *T*_min_ = 0.980, *T*_max_ = 0.985
5731 measured reflections
2746 independent reflections
2155 reflections with *I* > 2σ(*I*)
*R*_int_ = 0.027

#### Refinement

*R*[*F* ^2^ > 2σ(*F* ^2^)] = 0.038
*wR*(*F* ^2^) = 0.109
*S* = 1.09
2746 reflections
239 parameters
4 restraints
H atoms treated by a mixture of independent and constrained refinement
Δρ_max_ = 0.20 e Å^−3^
Δρ_min_ = −0.25 e Å^−3^

### 

Data collection: *SMART* (Bruker, 1998 ▶); cell refinement: *SAINT* (Bruker, 1998 ▶); data reduction: *SAINT*; program(s) used to solve structure: *SHELXS97* (Sheldrick, 2008 ▶); program(s) used to refine structure: *SHELXL97* (Sheldrick, 2008 ▶); molecular graphics: *SHELXTL* (Sheldrick, 2008 ▶); software used to prepare material for publication: *SHELXTL*.

## Supplementary Material

Crystal structure: contains datablocks global, I. DOI: 10.1107/S1600536810052062/hb5769sup1.cif

Structure factors: contains datablocks I. DOI: 10.1107/S1600536810052062/hb5769Isup2.hkl

Additional supplementary materials:  crystallographic information; 3D view; checkCIF report

## Figures and Tables

**Table 1:** Hydrogen-bond geometry (Å, °)

*D*—H⋯*A*	*D*—H	H⋯*A*	*D*⋯*A*	*D*—H⋯*A*
O1—H1⋯O2^i^	0.82	1.89	2.7003 (17)	171
O2—H2*A*⋯N3^ii^	0.88 (1)	1.91 (1)	2.7655 (16)	167 (2)
N2—H2*C*⋯O2^ii^	0.91 (1)	2.09 (1)	2.9715 (19)	164 (2)
O2—H2*B*⋯N4^iii^	0.87 (1)	1.99 (1)	2.8254 (17)	162 (2)

Symmetry codes: (i) 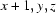; (ii) 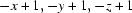; (iii) 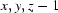.

**Fig. 1.**
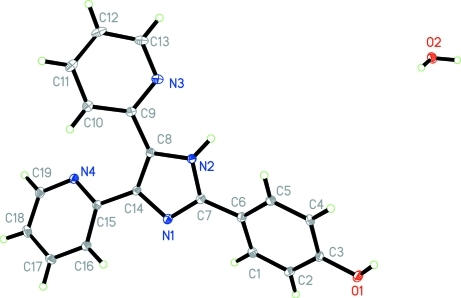
The molecular structure of the title compound, showing 30% probability displacement ellipsoids for the non-hydrogen atoms.

**Fig. 2.**
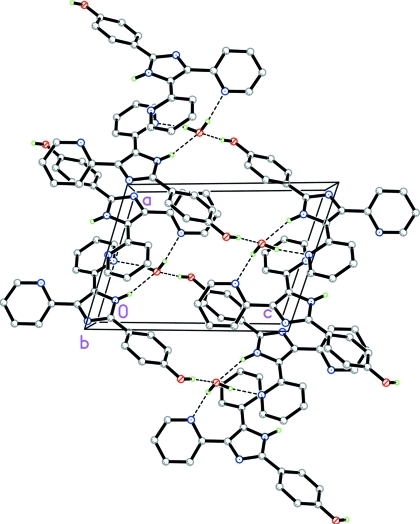
The packing diagram of the title compound. Hydrogen bonds are shown as dashed lines.

## supplementary crystallographic information

### Comment

Lophine, 2,4,5-triphenylimidazole, is a well known potential chemiluminesscent (CL) compound (Radziszewsky, 1877). 2-(4-Hydroxyphenyl)-4,5-di(2-pyridyl)imidazole was synthesized by the methods similar to those previously reported (Nakashima *et al.*, 1995; Kuroda *et al.*, 1993). Recently, we have synthesized an analogic structure of imidazole derivative, namely, the title compound, 2-(4-hydroxyphenyl)-4,5-di(2-pyridyl)imidazole. We present its crystal structure here.

The compound consists of a 2-(4-hydroxyphenyl)-4,5-di(2-pyridyl)imidazole molecule and a water molecule of crystallization (Fig. 1). The central imidazole ring forms dihedral angles of 25.3 (2), 22.5 (2), and 29.2 (2)°, respectively, with the C1—C6 benzene ring, C9—C13/N3 pyridine ring, and C15—C19/N4 pyridine ring. The dihedral angle between the two pyridine rings is 38.0 (2)°. The dihedral angle between the central imidazole ring and the benzene ring is 25.3 (2)°. The crystal structure is stabilized by intermolecular O—H···O, O—H···N, and N—H···O hydrogen bonds (Fig. 2, and Table 1).

### Experimental

The title compound was prepared by the reaction of 2, 2'-pyridyl (1.0 mmol), 4-hydroxybenzaldehyde (1.0 mmol) and ammonium acetate (10 mmol) in 8 ml acetic acid refluxed for 6 h. After cooling to room temperature, the mixture was poured into water, the precipitate was filtered off and dried to give the target compound in 20% yield. Colourless prisms of the title compound were grown by slow evaporation of a solution in mathanol.

### Refinement

H2A, H2B, and H2C atoms were located in a difference Fourier map, with N—H, O—H and H···H distances restrained to 0.90 (1), 0.85 (1), and 1.45 (2) Å, respectively. The remaining H atoms were placed in calculated positions (C—H = 0.93 Å) and refined as riding with *U*_iso_(H) = 1.2*U*_eq_(C).

### Crystal data

**Table:**

C_19_H_14_N_4_O·H_2_O	*Z* = 2
*M**_r_* = 332.36	*F*(000) = 348
Triclinic, *P*1	*D*_x_ = 1.404 Mg m^−^^3^
Hall symbol: -P 1	Mo *K*α radiation, λ = 0.71073 Å
*a* = 8.5875 (17) Å	Cell parameters from 2376 reflections
*b* = 9.0151 (18) Å	θ = 2.6–27.9°
*c* = 11.353 (2) Å	µ = 0.10 mm^−^^1^
α = 77.89 (3)°	*T* = 113 K
β = 69.96 (3)°	Prism, colourless
γ = 73.66 (3)°	0.22 × 0.20 × 0.16 mm
*V* = 786.0 (3) Å^3^	

### Data collection

**Table:**

Bruker SMART CCD diffractometer	2746 independent reflections
Radiation source: fine-focus sealed tube	2155 reflections with *I* > 2σ(*I*)
graphite	*R*_int_ = 0.027
ω scans	θ_max_ = 25.0°, θ_min_ = 2.6°
Absorption correction: multi-scan (*SADABS*; Bruker, 1998)	*h* = −10→10
*T*_min_ = 0.980, *T*_max_ = 0.985	*k* = −10→10
5731 measured reflections	*l* = −13→12

### Refinement

**Table:**

Refinement on *F*^2^	Primary atom site location: structure-invariant direct methods
Least-squares matrix: full	Secondary atom site location: difference Fourier map
*R*[*F*^2^ > 2σ(*F*^2^)] = 0.038	Hydrogen site location: inferred from neighbouring sites
*wR*(*F*^2^) = 0.109	H atoms treated by a mixture of independent and constrained refinement
*S* = 1.09	*w* = 1/[σ^2^(*F*_o_^2^) + (0.0736*P*)^2^] where *P* = (*F*_o_^2^ + 2*F*_c_^2^)/3
2746 reflections	(Δ/σ)_max_ = 0.001
239 parameters	Δρ_max_ = 0.20 e Å^−^^3^
4 restraints	Δρ_min_ = −0.25 e Å^−^^3^

### Special details

**Table:**

Geometry. All e.s.d.'s (except the e.s.d. in the dihedral angle between two l.s. planes) are estimated using the full covariance matrix. The cell e.s.d.'s are taken into account individually in the estimation of e.s.d.'s in distances, angles and torsion angles; correlations between e.s.d.'s in cell parameters are only used when they are defined by crystal symmetry. An approximate (isotropic) treatment of cell e.s.d.'s is used for estimating e.s.d.'s involving l.s. planes.
Refinement. Refinement of *F*^2^ against ALL reflections. The weighted *R*-factor *wR* and goodness of fit *S* are based on *F*^2^, conventional *R*-factors *R* are based on *F*, with *F* set to zero for negative *F*^2^. The threshold expression of *F*^2^ > σ(*F*^2^) is used only for calculating *R*-factors(gt) *etc*. and is not relevant to the choice of reflections for refinement. *R*-factors based on *F*^2^ are statistically about twice as large as those based on *F*, and *R*- factors based on ALL data will be even larger.

### Fractional atomic coordinates and isotropic or equivalent isotropic displacement parameters (Å^2^)

**Table:**

	*x*	*y*	*z*	*U*_iso_*/*U*_eq_	
O1	1.35444 (13)	0.11009 (12)	0.44275 (9)	0.0221 (3)	
H1	1.3597	0.1807	0.3838	0.033*	
N1	0.95455 (15)	0.27226 (13)	1.01220 (10)	0.0162 (3)	
N2	0.80962 (15)	0.46738 (13)	0.90993 (10)	0.0152 (3)	
N3	0.52123 (15)	0.70299 (13)	0.99216 (10)	0.0187 (3)	
N4	0.68226 (15)	0.36680 (13)	1.32116 (10)	0.0175 (3)	
C1	1.12180 (18)	0.11950 (15)	0.77615 (12)	0.0175 (3)	
H1A	1.1067	0.0502	0.8503	0.021*	
C2	1.22861 (18)	0.06627 (16)	0.66424 (12)	0.0179 (3)	
H2	1.2854	−0.0381	0.6633	0.022*	
C3	1.25179 (18)	0.16872 (16)	0.55213 (12)	0.0160 (3)	
C4	1.16603 (18)	0.32453 (16)	0.55465 (12)	0.0173 (3)	
H4	1.1808	0.3935	0.4804	0.021*	
C5	1.05921 (18)	0.37679 (16)	0.66722 (12)	0.0174 (3)	
H5	1.0018	0.4810	0.6679	0.021*	
C6	1.03582 (17)	0.27565 (15)	0.78036 (12)	0.0155 (3)	
C7	0.93382 (17)	0.33457 (15)	0.90045 (12)	0.0154 (3)	
C8	0.74579 (17)	0.49413 (15)	1.03485 (12)	0.0144 (3)	
C9	0.61392 (17)	0.63503 (15)	1.07191 (12)	0.0153 (3)	
C10	0.58923 (18)	0.69920 (15)	1.18034 (13)	0.0187 (3)	
H10	0.6575	0.6535	1.2325	0.022*	
C11	0.46215 (19)	0.83145 (16)	1.20923 (14)	0.0232 (3)	
H11	0.4441	0.8763	1.2809	0.028*	
C12	0.3623 (2)	0.89615 (17)	1.13019 (15)	0.0272 (4)	
H12	0.2730	0.9828	1.1493	0.033*	
C13	0.39737 (19)	0.83010 (16)	1.02306 (14)	0.0244 (4)	
H13	0.3318	0.8759	0.9689	0.029*	
C14	0.83707 (18)	0.37066 (15)	1.09693 (12)	0.0145 (3)	
C15	0.83185 (18)	0.32729 (15)	1.23068 (12)	0.0151 (3)	
C16	0.97916 (18)	0.24099 (15)	1.25986 (12)	0.0174 (3)	
H16	1.0801	0.2143	1.1956	0.021*	
C17	0.97453 (19)	0.19538 (16)	1.38436 (13)	0.0203 (3)	
H17	1.0713	0.1362	1.4053	0.024*	
C18	0.8229 (2)	0.23941 (16)	1.47772 (13)	0.0220 (3)	
H18	0.8166	0.2130	1.5627	0.026*	
C19	0.68134 (19)	0.32344 (16)	1.44204 (12)	0.0202 (3)	
H19	0.5796	0.3516	1.5053	0.024*	
O2	0.39964 (13)	0.34896 (12)	0.25546 (9)	0.0233 (3)	
H2A	0.439 (2)	0.337 (3)	0.1750 (10)	0.068 (7)*	
H2B	0.473 (2)	0.374 (2)	0.2803 (15)	0.066 (7)*	
H2C	0.764 (2)	0.5192 (18)	0.8472 (12)	0.042 (5)*	

### Atomic displacement parameters (Å^2^)

**Table:**

	*U*^11^	*U*^22^	*U*^33^	*U*^12^	*U*^13^	*U*^23^
O1	0.0213 (6)	0.0233 (6)	0.0160 (5)	−0.0012 (4)	−0.0004 (4)	−0.0043 (4)
N1	0.0174 (7)	0.0154 (6)	0.0144 (6)	−0.0033 (5)	−0.0028 (5)	−0.0028 (5)
N2	0.0153 (7)	0.0152 (6)	0.0131 (6)	−0.0023 (5)	−0.0035 (5)	−0.0010 (5)
N3	0.0157 (7)	0.0175 (6)	0.0214 (6)	−0.0030 (5)	−0.0060 (5)	0.0004 (5)
N4	0.0180 (7)	0.0177 (6)	0.0151 (6)	−0.0027 (5)	−0.0039 (5)	−0.0027 (5)
C1	0.0196 (8)	0.0167 (7)	0.0160 (7)	−0.0056 (6)	−0.0056 (6)	0.0006 (6)
C2	0.0164 (8)	0.0150 (7)	0.0215 (7)	−0.0023 (6)	−0.0038 (6)	−0.0054 (6)
C3	0.0141 (7)	0.0204 (7)	0.0141 (7)	−0.0045 (6)	−0.0026 (6)	−0.0057 (6)
C4	0.0207 (8)	0.0189 (7)	0.0128 (7)	−0.0061 (6)	−0.0059 (6)	0.0006 (5)
C5	0.0199 (8)	0.0139 (7)	0.0184 (7)	−0.0025 (6)	−0.0065 (6)	−0.0028 (5)
C6	0.0141 (7)	0.0177 (7)	0.0159 (7)	−0.0057 (6)	−0.0041 (6)	−0.0027 (6)
C7	0.0154 (8)	0.0142 (7)	0.0166 (7)	−0.0041 (6)	−0.0038 (6)	−0.0024 (6)
C8	0.0140 (7)	0.0158 (7)	0.0131 (7)	−0.0050 (6)	−0.0021 (6)	−0.0022 (5)
C9	0.0130 (7)	0.0142 (7)	0.0168 (7)	−0.0053 (6)	−0.0016 (6)	0.0003 (5)
C10	0.0184 (8)	0.0184 (7)	0.0185 (7)	−0.0061 (6)	−0.0036 (6)	−0.0011 (6)
C11	0.0230 (8)	0.0176 (7)	0.0245 (8)	−0.0049 (6)	0.0009 (6)	−0.0063 (6)
C12	0.0187 (8)	0.0173 (7)	0.0375 (9)	0.0024 (6)	−0.0018 (7)	−0.0064 (7)
C13	0.0180 (8)	0.0203 (8)	0.0317 (8)	−0.0009 (6)	−0.0092 (7)	0.0015 (6)
C14	0.0129 (7)	0.0150 (7)	0.0150 (7)	−0.0034 (5)	−0.0025 (6)	−0.0034 (5)
C15	0.0171 (8)	0.0119 (7)	0.0166 (7)	−0.0039 (5)	−0.0045 (6)	−0.0030 (5)
C16	0.0179 (8)	0.0145 (7)	0.0193 (7)	−0.0030 (6)	−0.0048 (6)	−0.0036 (6)
C17	0.0230 (8)	0.0166 (7)	0.0245 (8)	−0.0040 (6)	−0.0129 (7)	−0.0004 (6)
C18	0.0304 (9)	0.0217 (8)	0.0168 (7)	−0.0085 (7)	−0.0104 (7)	0.0005 (6)
C19	0.0230 (9)	0.0208 (8)	0.0145 (7)	−0.0053 (6)	−0.0024 (6)	−0.0022 (6)
O2	0.0195 (6)	0.0314 (6)	0.0179 (6)	−0.0060 (5)	−0.0058 (4)	−0.0005 (5)

### Geometric parameters (Å, °)

**Table:**

O1—C3	1.3644 (17)	C8—C14	1.382 (2)
O1—H1	0.8200	C8—C9	1.4702 (19)
N1—C7	1.3252 (17)	C9—C10	1.3950 (19)
N1—C14	1.3854 (18)	C10—C11	1.381 (2)
N2—C7	1.3563 (18)	C10—H10	0.9300
N2—C8	1.3807 (16)	C11—C12	1.381 (2)
N2—H2C	0.908 (9)	C11—H11	0.9300
N3—C13	1.3397 (19)	C12—C13	1.370 (2)
N3—C9	1.3473 (18)	C12—H12	0.9300
N4—C19	1.3436 (17)	C13—H13	0.9300
N4—C15	1.3505 (18)	C14—C15	1.4747 (18)
C1—C2	1.3767 (19)	C15—C16	1.393 (2)
C1—C6	1.3958 (19)	C16—C17	1.3767 (18)
C1—H1A	0.9300	C16—H16	0.9300
C2—C3	1.3961 (19)	C17—C18	1.384 (2)
C2—H2	0.9300	C17—H17	0.9300
C3—C4	1.392 (2)	C18—C19	1.381 (2)
C4—C5	1.3794 (19)	C18—H18	0.9300
C4—H4	0.9300	C19—H19	0.9300
C5—C6	1.3986 (19)	O2—H2A	0.876 (9)
C5—H5	0.9300	O2—H2B	0.870 (9)
C6—C7	1.4601 (18)		
			
C3—O1—H1	109.5	C10—C9—C8	122.56 (12)
C7—N1—C14	105.45 (12)	C11—C10—C9	119.19 (14)
C7—N2—C8	108.44 (11)	C11—C10—H10	120.4
C7—N2—H2C	124.9 (11)	C9—C10—H10	120.4
C8—N2—H2C	125.9 (11)	C10—C11—C12	119.02 (14)
C13—N3—C9	118.20 (12)	C10—C11—H11	120.5
C19—N4—C15	117.30 (12)	C12—C11—H11	120.5
C2—C1—C6	121.15 (13)	C13—C12—C11	118.64 (14)
C2—C1—H1A	119.4	C13—C12—H12	120.7
C6—C1—H1A	119.4	C11—C12—H12	120.7
C1—C2—C3	120.07 (13)	N3—C13—C12	123.43 (14)
C1—C2—H2	120.0	N3—C13—H13	118.3
C3—C2—H2	120.0	C12—C13—H13	118.3
O1—C3—C4	122.37 (12)	C8—C14—N1	110.41 (12)
O1—C3—C2	118.14 (12)	C8—C14—C15	132.74 (13)
C4—C3—C2	119.45 (13)	N1—C14—C15	116.85 (12)
C5—C4—C3	120.07 (12)	N4—C15—C16	122.02 (12)
C5—C4—H4	120.0	N4—C15—C14	118.81 (13)
C3—C4—H4	120.0	C16—C15—C14	119.11 (13)
C4—C5—C6	121.06 (13)	C17—C16—C15	119.66 (14)
C4—C5—H5	119.5	C17—C16—H16	120.2
C6—C5—H5	119.5	C15—C16—H16	120.2
C1—C6—C5	118.19 (12)	C16—C17—C18	118.66 (14)
C1—C6—C7	121.05 (12)	C16—C17—H17	120.7
C5—C6—C7	120.62 (12)	C18—C17—H17	120.7
N1—C7—N2	111.15 (12)	C19—C18—C17	118.60 (13)
N1—C7—C6	125.50 (13)	C19—C18—H18	120.7
N2—C7—C6	123.27 (12)	C17—C18—H18	120.7
N2—C8—C14	104.55 (12)	N4—C19—C18	123.71 (13)
N2—C8—C9	120.20 (11)	N4—C19—H19	118.1
C14—C8—C9	135.20 (12)	C18—C19—H19	118.1
N3—C9—C10	121.40 (13)	H2A—O2—H2B	111.3 (13)
N3—C9—C8	116.00 (11)		
			
C6—C1—C2—C3	−0.4 (2)	C14—C8—C9—C10	21.5 (2)
C1—C2—C3—O1	−177.92 (12)	N3—C9—C10—C11	2.8 (2)
C1—C2—C3—C4	−0.1 (2)	C8—C9—C10—C11	−179.71 (12)
O1—C3—C4—C5	177.80 (12)	C9—C10—C11—C12	0.3 (2)
C2—C3—C4—C5	0.0 (2)	C10—C11—C12—C13	−2.5 (2)
C3—C4—C5—C6	0.4 (2)	C9—N3—C13—C12	1.3 (2)
C2—C1—C6—C5	0.8 (2)	C11—C12—C13—N3	1.8 (2)
C2—C1—C6—C7	−174.87 (12)	N2—C8—C14—N1	0.65 (14)
C4—C5—C6—C1	−0.9 (2)	C9—C8—C14—N1	−176.67 (13)
C4—C5—C6—C7	174.85 (12)	N2—C8—C14—C15	−179.37 (13)
C14—N1—C7—N2	0.11 (14)	C9—C8—C14—C15	3.3 (3)
C14—N1—C7—C6	176.81 (12)	C7—N1—C14—C8	−0.48 (14)
C8—N2—C7—N1	0.29 (15)	C7—N1—C14—C15	179.53 (11)
C8—N2—C7—C6	−176.50 (11)	C19—N4—C15—C16	1.93 (18)
C1—C6—C7—N1	24.1 (2)	C19—N4—C15—C14	179.00 (11)
C5—C6—C7—N1	−151.45 (13)	C8—C14—C15—N4	30.9 (2)
C1—C6—C7—N2	−159.53 (12)	N1—C14—C15—N4	−149.14 (12)
C5—C6—C7—N2	24.87 (19)	C8—C14—C15—C16	−151.97 (15)
C7—N2—C8—C14	−0.56 (14)	N1—C14—C15—C16	28.01 (17)
C7—N2—C8—C9	177.25 (11)	N4—C15—C16—C17	−0.8 (2)
C13—N3—C9—C10	−3.58 (19)	C14—C15—C16—C17	−177.87 (11)
C13—N3—C9—C8	178.78 (11)	C15—C16—C17—C18	−1.13 (19)
N2—C8—C9—N3	22.10 (17)	C16—C17—C18—C19	1.86 (19)
C14—C8—C9—N3	−160.90 (14)	C15—N4—C19—C18	−1.17 (19)
N2—C8—C9—C10	−155.51 (12)	C17—C18—C19—N4	−0.7 (2)

### Hydrogen-bond geometry (Å, °)

**Table:**

*D*—H···*A*	*D*—H	H···*A*	*D*···*A*	*D*—H···*A*
O1—H1···O2^i^	0.82	1.89	2.7003 (17)	171
O2—H2A···N3^ii^	0.88 (1)	1.91 (1)	2.7655 (16)	167.(2)
N2—H2C···O2^ii^	0.91 (1)	2.09 (1)	2.9715 (19)	164.(2)
O2—H2B···N4^iii^	0.87 (1)	1.99 (1)	2.8254 (17)	162.(2)

Symmetry codes: (i) *x*+1, *y*, *z*; (ii) −*x*+1, −*y*+1, −*z*+1; (iii) *x*, *y*, *z*−1.

## References

[bb1] Bruker (1998). *SMART*, *SAINT* and *SADABS* Bruker AXS Inc., Madison, Wisconsin, USA.

[bb2] Kuroda, N., Takatani, M., Nakashima, K., Akiyama, S. & Ohkura, Y. (1993). *Biol. Pharm. Bull.* **16**, 220–222. 10.1248/bpb.16.220 8364463

[bb3] Nakashima, K., Yamasaki, H., Kuroda, N. & Akiyama, S. (1995). *Anal. Chim. Acta*, **303**, 103–107.

[bb4] Radziszewsky, B. (1877). *Chem. Ber.* **10**, 70–75.

[bb5] Sheldrick, G. M. (2008). *Acta Cryst.* A**64**, 112–122. 10.1107/S0108767307043930 18156677

